# Validation of an web-based dietary assessment tool (RiksmatenFlex) against doubly labelled water and 24 h dietary recalls in pregnant women

**DOI:** 10.1186/s12937-024-00987-5

**Published:** 2024-07-30

**Authors:** Emmie Söderström, Johanna Sandborg, Ellinor Nilsson, Maria Henström, Eva Warensjö Lemming, Anna Karin Lindroos, Jennifer Rood, Jessica Petrelius Sipinen, Marie Löf

**Affiliations:** 1 Department of Health, Medicine and Caring Sciences, Linköping University, Linköping, Sweden; 2 Department of Medicine, Karolinska Institutet, Huddinge, Sweden; 3 Medical Epidemiology, Department of Surgical Sciences, Uppsala University, Uppsala, Sweden; 4 Department of Food science, Nutrition and Dietetics, Uppsala University, Uppsala, Sweden; 5 Swedish Food Agency, Uppsala, Sweden; 6 Department of Internal Medicine and Clinical Nutrition, the Sahlgrenska Academy, University of Gothenburg, Gothenburg, Sweden; 7 Pennington Biomedical Research Center, Baton Rouge, LA USA

**Keywords:** Dietary assessments, Web-based tools, Doubly labelled water, 24 h recalls

## Abstract

**Introduction:**

Digital technologies have enabled new possibilities to assess dietary intake and have shown promise in terms of decreased participant burden, improved accuracy and lower costs. However, their potential and validity in pregnant populations are scarcely explored.

**Objectives:**

This study aimed to (a) validate energy intakes obtained from a web-based dietary recall method developed for national surveys (RiksmatenFlex) against total energy expenditure (TEE) by means of the doubly labelled water (DLW) method, and (b) to compare intakes of macronutrients, key unhealthy and healthy foods as well as adherence to food-based dietary guidelines between RiksmatenFlex and repeated 24 h telephone dietary recalls in healthy Swedish pregnant women.

**Methods:**

This study was conducted as a nested validation within the HealthyMoms trial. Intakes of foods, macronutrients and energy were assessed during three days through RiksmatenFlex and 24 h telephone dietary recalls, and Swedish Healthy Eating Index (SHEI) scores were also calculated for both methods (*n* = 52). For 24 women, TEE was also assessed through the DLW method. Paired Samples T-tests and Wilcoxon Signed Ranks Tests were used to identify differences between means for foods, macronutrients, energy and SHEI scores. Pearson correlation coefficient or Spearman’s rho were performed to identify relationships between variables. To compare energy intake (RiksmatenFlex) with TEE (DLW method) and 24 h telephone dietary recalls, Bland and Altman plots were constructed.

**Results:**

Average energy intake from RiksmatenFlex (10,015 [SD 2004] kJ) was not statistically different from TEE (10,252 [SD 1197] kJ) (*p* = 0.596) (mean difference: -237 kJ/24 h). Correspondingly, there were small mean differences between average intakes of key unhealthy and healthy foods and average SHEI scores between RiksmatenFlex and 24 h telephone dietary recalls. However, the Bland and Altman plots showed wide limits of agreement for all dietary variables (e.g., for energy intake using RiksmatenFlex versus TEE: ±4239 kJ/24 h). High correlations between the investigated dietary variables for the two dietary methods were observed (*r* = 0.751 to 0.931; all *p* < 0.001).

**Conclusion:**

RiksmatenFlex captured average intakes of energy, unhealthy and healthy food groups and adherence to food-based dietary guidelines in a comparable way to 24 h telephone dietary recalls and the DLW method. Our results support the validity of RiksmatenFlex as a web-based dietary assessment method for future use in pregnancy for intervention studies and national dietary surveys.

**Supplementary Information:**

The online version contains supplementary material available at 10.1186/s12937-024-00987-5.

## Introduction

Pregnancy is a critical period where maternal lifestyle behaviors such as dietary habits have significant impact on health outcomes in both the pregnant woman and the growing child. For instance, a healthy diet has been associated with lower risk for gestational diabetes mellitus [1], large-for-gestational-age infants [1], excessive gestational weight gain (GWG) [1–3], and having a better maternal cardiometabolic health in pregnancy [4]. Given the increased prevalence of obesity and excessive GWG in pregnant women in many high-income countries [5–7], dietary intakes are clearly not optimal. Correspondingly, a recent meta-analysis (54 studies, *n* = 135 566 pregnant women, several world regions) concluded that suboptimal energy intakes were common and requested more efforts to encourage women to adopt healthy eating habits during pregnancy [8]. Validity of dietary assessment methods plays a vital role in accurately determining dietary intakes and adherence to recommendations as well as assessing dietary intervention effects. Therefore, access to scalable and accurate methods to assess dietary intake in pregnant women is essential. This is particularly important for methods which are intended to be used at a large scale to monitor dietary intakes in the general population, including pregnant women.

To date, there is no reference method with high accuracy and precision to assess diet and most methods (e.g., food frequency questionnaires, dietary history and weighed diet records) have been reported to have limited accuracy with predominantly under-reporting of energy intake being common for all methods [9–11]. Moreover, these traditional approaches are often both time consuming and burdensome for participants and researchers to perform [12]. With digitalisation, various technology-assisted methods to assess dietary intake have become increasingly available, including web-based tools, food photography images or wearable cameras. These methods can be advantageous by facilitating access, be less time consuming for participants to use, could increase accuracy of reported intakes [13] and can be used in studies with large sample sizes. One such example is the web-based dietary recall method RiksmatenFlex [14] which was developed by the Swedish Food Agency for their regular national dietary surveys in different age groups and it has also been used in research studies (e.g., the HealthyMoms trial) [15]. RiksmatenFlex consists of a flexible online registration platform supporting both prospective and retrospective dietary recordings, e.g., repeated 24 h dietary recalls, which also can be adapted to various study populations. Although RiksmatenFlex has been validated in adolescents [13], and is currently being validated in young children [16], it is yet to be validated in a pregnant population. This is important since the type and amount of food consumed might differ in this group [17, 18], and there might also be differences in the accuracy of reporting. Moreover, to the best of our knowledge, no previous study has assessed the validity of a web-based and scalable dietary recall method in pregnant women compared to traditional data collection approaches (i.e., telephone-based 24 h dietary recalls) and the doubly labelled water (DLW) method, which is the reference method to validate energy intake [16].

### Objectives

The aims of this nested validation study within the HealthyMoms trial [15, 19] were (a) to validate energy intakes obtained from RiksmatenFlex against TEE by means of the DLW method, and (b) to compare intakes of macronutrients, key unhealthy and healthy foods as well as adherence to food-based dietary guidelines (the Swedish Healthy Eating Index, SHEI, score) between RiksmatenFlex and three repeated 24 h telephone dietary recalls in healthy Swedish pregnant women.

## Materials and methods

### Participants and study design

**Fig. 1:**
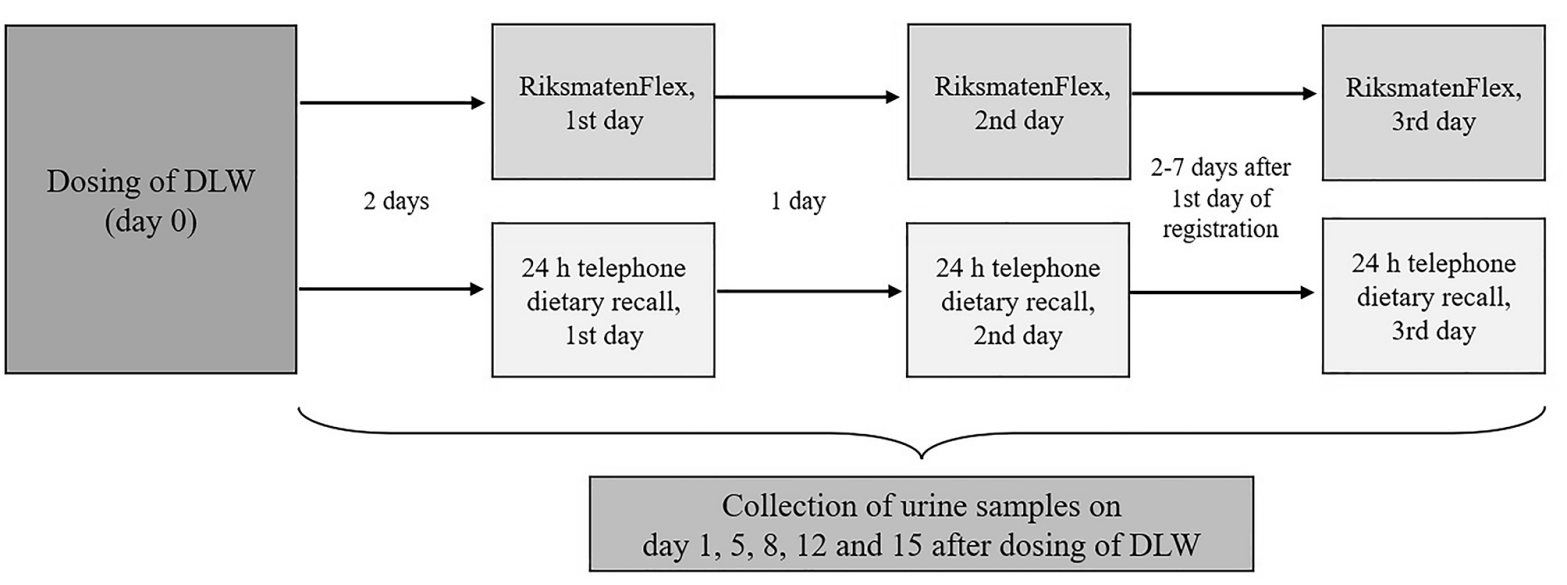
Study overview showing dosing of doubly labelled water (DLW) and timing of RiksmatenFlex and 24 h telephone dietary recalls

This validation study was conducted with a sub-sample of pregnant women participating in the HealthyMoms trial (*n* = 305) [15] which took place in Östergötland county, Sweden. The trial targeted pregnant women and aimed to evaluate an app (HealthyMoms) as a support for healthy weight gain, dietary- and physical activity habits. Recruitment and study population have previously been described in detail [15]. During the final recruitment phase of the HealthyMoms trial (October 2019 to March 2020), we performed this validation of energy- and dietary intake obtained from RiksmatenFlex (part of the baseline assessment in the trial). To validate dietary intake, we conducted three repeated telephone interviews, i.e., 24 h telephone dietary recalls, with women for the same days that they performed an online registration of their diet, i.e., RiksmatenFlex (*n* = 52). For validation of energy intake obtained with RiksmatenFlex, participants were also invited to participate in a validation of their energy intakes using the DLW method and 24 women accepted to do so. Figure 1 shows an overview of the study design including the timing of the three methods. In short, participants started by drinking DLW and were given instructions to log into RiksmatenFlex two days after to register their diet. The research team then matched the 24 h telephone dietary recalls with the days that participants registered their diet in RiksmatenFlex. Data for this nested validation study was collected between October 2019 and March 2020 and all data was collected prior to randomization in the trial. There were no major differences in baseline characteristics for women participating in the validation study (*n* = 52 and *n* = 24) compared to all women in the HealthyMoms trial (*n* = 305) (see results section). All participants provided their written informed consents, and all procedures were approved by the Regional Ethical Review Board in Linköping (reference numbers 2017/112 − 31, and 2018/262 − 32;).

### Methods

#### RiksmatenFlex

All participants were instructed to use the web-based dietary assessment tool RiksmatenFlex, developed by the Swedish Food Agency [14]. The assessment is based on repeated (3 days) 24 h dietary recalls, where participants reported the first two days consecutively whereas the third day was randomly assigned 2–7 days after the first day to ensure that both week- and weekend days were covered [20]. Through the 24 h dietary recalls, participants recalled all intake of food and drinks. To exemplify, when logging in to the system for the first time, participants were asked to register all their intake of foods and drinks from the day before. Amounts of registered items were specified through household measures (e.g., dl and tablespoons for foods such as yoghurt) or slices/pieces (e.g., for bread) and for some foods and complete dishes, pictures of various portion sizes too chose from were provided. The registered intakes of foods and drinks were linked with the Swedish Food Agency Food composition database [21] to provide daily intakes of energy and macronutrients for each participant. Registrations with daily energy intake that exceeded 3500 kcal or were less than 800 kcal were manually checked to ensure that no obvious incorrect imputations had been made, e.g., incomplete registrations or any mistakes regarding amounts or other evident misunderstandings [20]. Two days exceeded 3500 kcal and were manually checked but deemed plausible and were thus not excluded from the analysis. Data on selected key food groups and drinks representing healthy and unhealthy foods including fruit and vegetables, fish and shellfish, red and processed meat, nuts and seeds, fruit juice, sugar sweetened beverages, artificial sweetened beverages, sweet and savoury treats (i.e., ice cream, candy, baked goods, desserts, chips and popcorn) were then summarised for each participant and day and a mean intake was calculated (in grams/day or week). Finally, for each participant, a SHEI score was calculated as a measure of compliance to existing Swedish food-based dietary guidelines [22] and recommendations for specific nutrients [23]. The score included the following components: fruit and vegetables (g/day), whole grain (g/10 MJ), fibre (g/MJ), polyunsaturated fatty acids (Energy %, E%), monounsaturated fatty acids (E%), saturated fatty acids (E%), fish and shellfish (g/day), red meat (g/week) and added sugar (E%) (Additional file, Supplementary Table 1). An individual score (0 to 1) was calculated for each component and these scores were then summarised into a total SHEI score (0 to 9), as previously been described [22]. High scores indicated better compliance to Swedish dietary recommendations.

#### 24 h telephone dietary recalls

All participants partook in telephone based 24 h dietary recalls matching the days for RiksmatenFlex, and completion order of the two methods was random, i.e., sometimes participants finished their registration in RiksmatenFlex before the 24 h telephone dietary recall and sometimes it was done afterwards. Participants were asked to report their intake of food and drinks the previous day and to specify amounts. Amounts were specified through measures such as deciliters, tablespoons and teaspoons. Other foods, e.g., bread and sweets, were specified in terms of slices or pieces. Based on reported intakes of foods and drinks, the interviewer asked follow-up questions to capture foods used in mixed dishes, cooking methods as well as e.g., fat and fiber content of certain foods. All dietary data was imputed into a software program (Dietist Net Pro, Kost och Näringsdata AB, Stockholm, Sweden) where it was linked to the Swedish food composition database [21], to provide data on intakes of energy, food groups and macronutrients. Similar to RiksmatenFlex, food groups (i.e., fruit and vegetables, fish and shellfish, red and processed meat, nuts and seeds, fruit juice, sugar sweetened beverages, artificial sweetened beverages, sweet and savoury treats, defined as above) were summarized for each participant and day and means were calculated (in grams/day or week). Correspondingly, a SHEI score for each participant was calculated from the 24 h telephone dietary recalls in the same way as described above for RiksmatenFlex.

#### Doubly labelled water

Participants that accepted to participate in the validation of energy intake (*n* = 24) were instructed to collect two baseline urine samples prior to coming to Linköping University hospital for an assessment of TEE in early pregnancy (12.6 ± 1.2 gestational weeks). Participants were weighed in light clothing and were given an accurately weighed dose of stable isotopes (0.08 g ^2^H_2_O and 0.15 g H_2_^18^O, per kg body weight). After drinking the water, participants were instructed to collect five urine samples during the following two weeks (on day 1, 5, 8, 12 and 15). Each sample was marked with a date and time for sampling. During the same two-week period, assessment of participant energy intake through RiksmatenFlex was performed, as previously described. Collected urine samples were stored (-18 °C) at Linköping University Hospital until analysis. Samples were shipped to, and analyzed at Pennington Biomedical Research Center, Baton Rouge, Louisiana, the United States.

Doses and urine samples were analyzed for ^18^O and ^2^H abundance by isotope ratio mass spectrometry using automated devices for deuterium (H/Device, Finnigan) and ^18^O (GasBench, Finnigan) [24]. The isotopic enrichments of the post-dose urine samples compared with the pre-dose samples were used to calculate elimination rates (k_d_ and k_O_) using linear regression. The quotient between the ^2^H dilution space and ^18^O dilution space was 1.035 ± 0.010 for the 24 women. The CO_2_ production was calculated using the equation of Speakman et al. [25]. TEE was calculated by multiplying CO_2_ production by the energy equivalent of CO_2_ based on the estimated food quotient of the diet (0.86) [26].

### Statistics and power considerations

Values for energy intake, TEE, key unhealthy and healthy foods, macronutrients, and SHEI score are presented as means and standard deviations (SD). Paired Samples T-tests and Wilcoxon Signed Ranks Tests were used to identify differences between means for parametric and non-parametric data and correlation tests (Pearson correlation coefficient or Spearman’s rho) were performed to identify relationships between variables. To compare energy intake (RiksmatenFlex) with TEE (DLW method) and 24 h telephone dietary recalls, Bland and Altman plots were constructed [27], where the difference between the methods (y) were plotted against the average of the two methods (x). The mean difference of the methods as well as limits of agreement (mean difference ± 2SD) were calculated. Linear regression was used to evaluate the relationship between x and y in the plot. Mean intakes of macronutrients and the specified key unhealthy and healthy foods from RiksmatenFlex and 24 h telephone dietary recalls were also compared according to Bland and Altman [27]. A significance level of 5% was used. All analyses were performed using IBM SPSS Statistics version 29. This validation study was performed as a nested sub-study for the HealthyMoms trial which was carefully dimensioned to address the primary trial outcomes [15]. We also considered sample size a priori for the research questions investigated in this validation study. For the comparison of mean values (e.g., energy intake versus TEE) we estimated that TEE was on average 10,000 +/- 1300 kJ, assuming a correlation between energy intake and TEE of 0.6. Thus, 24 women would provide us with more than 80% power to detect a 7% difference in energy intake and TEE, which is considered as acceptable agreement in accordance with Lombard et al. [28]. A slightly higher target sample size for the comparison against 24 h telephone dietary recalls was decided (at least *n* = 50) which would provide us with the possibility to detect correlation coefficients between variables from the two methods from approximately 0.39 and higher, with lower correlation coefficients not deemed relevant.

## Results

Participant characteristics are presented in Table 1. No major differences were found for demographic data, anthropometrics or lifestyle behaviors in the DLW sub-sample (*n* = 24) and 24 h telephone dietary recall sub-sample (*n* = 52) compared to the entire HealthyMoms population (*n* = 305), respectively, except for slightly lower levels of moderate-to-vigorous physical activity in the two sub-samples for the validation. The compliance for the two dietary methods (*n* = 52) was high, with 48 women (92.3%) having three days of dietary data while four women (7.7%) had two days of registrations for both methods. All 24 women that were assessed using DLW had three days of recordings for both methods. In total, there were 152 days of data included in the analysis from RiksmatenFlex and 24 h telephone dietary recalls, respectively.

**Table 1:** Characteristics for study population and the entire HealthyMoms trial

	24 h recall subsample *n* = 52	DLW subsample *n* = 24^a^	HealthyMoms trial *n* = 305
Characteristics, n (%)			
Parity			
0	31 (59.6)	14 (58.3)	175 (57.4)
1 or more	21 (40.4)	10 (41.7)	130 (42.6)
University degree^b^	39 (75.0)	20 (83.3)	237 (77.7)
Pre-pregnancy BMI			
Underweight	2 (3.8)	0	6 (2.0)
Normal weight	36 (69.2)	18 (75.0)	212 (69.5)
Overweight	13 (25.0)	6 (25.0)	67 (22.0)
Obesity	1 (1.9)	0	20 (6.6)
Variables measured at baseline, mean (SD)			
Gestational week	14.0 (0.6)	14.1 (0.5)	13.9 (0.7)
Age (years)	31.7 (4.8)	31.8 (4.8)	31.3 (4.1)
Weight (kg)	65.5 (9.0)	67.1 (7.6)	67.7 (11.5)
Height (m)	1.67 (0.1)	1.69 (0.1)	1.67 (0.1)
BMI (kg/m^2^)	23.5 (2.9)	23.6 (2.5)	24.2 (3.8)
SHEI score	6.6 (1.0)	6.7 (1.0)	6.66 (0.98)^c^
MVPA (min/day)	33.2 (20.6)^d^	33.2 (23.5)	39.2 (24.0)^e^

BMI, Body Mass Index; MVPA, moderate-to-vigorous physical activity; SHEI, Swedish Healthy Eating Index

^a^ These women were also included in the 24 h recall sample

^b^ 24 h recall, *n* = 51; DLW, *n* = 23; HealthyMoms trial, *n* = 304

^c^*n* = 302 in the HealthyMoms trial for dietary intake

^d^*n* = 51

^e^*n* = 296

**Fig. 2:**
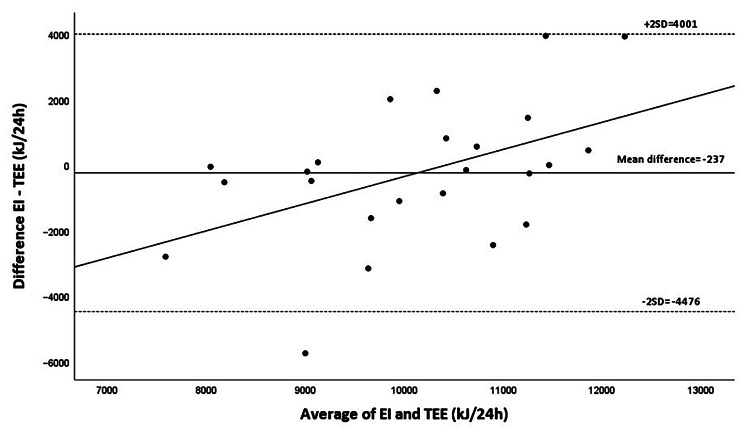
Bland and Altman plot for comparison of energy intake (EI) estimated using RiksmatenFlex compared to total energy expenditure (TEE) assessed through the doubly labelled water method. The mean difference between the methods was − 237 kJ/24 h and limits of agreement (2SD) of 4239 kJ/24 h. The regression equation was *y* = 0.83X – 8647 (*r* = 0.479, *p* = 0.018)

The average energy intake calculated using RiksmatenFlex (10,015 [SD 2004] kJ) was not statistically different from TEE measured with the DLW method (10,252 [SD 1197] kJ) (*P* = 0.596). The Bland and Altman plot (Fig. 2) displays energy intake from RiksmatenFlex and TEE from DLW. Although the mean difference was small (-237 kJ/24 h, -2.3% of average TEE), the limits of agreement were wide (± 4239 kJ/24 h) and there was a positive association between the difference and average of the two methods (*y* = 0.83X – 8647; *r* = 0.479, *P* = 0.018). More specifically, lower energy intake levels tended to be underestimated compared to TEE while higher levels tended to be overestimated.

**Fig. 3:**
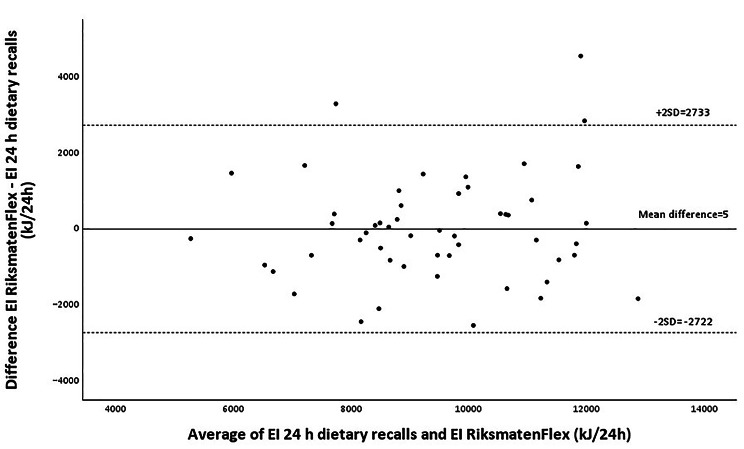
Bland and Altman plot for comparison of energy intake (EI) estimated using RiksmatenFlex compared to EI assessed through 24 h telephone dietary recalls. The mean difference between the methods was 5.3 kJ/24 h and limits of agreement (2SD) of 2727 kJ/24 h. The regression equation was *y* = 0.07x – 690 (*r* = 0.09, *p* = 0.515)

Table 2 shows average intakes and correlations for energy, macronutrients and key unhealthy and healthy foods estimated through RiksmatenFlex versus 24 h telephone dietary recalls. There was a significant positive correlation for energy intake between the two methods (*r* = 0.727, *P* < 0.001) and no significant difference between means was observed (9427 [SD 1817] kJ vs. 9432 [SD 1936] kJ, *P* = 0.978). The Bland and Altman plot showed large limits of agreement (± 2727 kJ/24 h) but there was no systematic trend in reports of energy intake using RiksmatenFlex compared to 24 h telephone dietary recalls (Fig. 3). Mean differences were low for all macronutrients (RiksmatenFlex vs. 24 h telephone dietary recalls: protein: -0.3, fat: -1.0 and carbohydrates: 1.2 E%, respectively) (Additional file, Supplementary Figures I-III). However, the Bland and Altman plots also showed wide limits of agreements corresponding to ± 3.7, ± 9.2 and ± 8.2 E%, respectively, with no proportional bias observed. Correspondingly, there were no statistically significant differences in average levels of key unhealthy and healthy foods between the two assessment methods (*P* = 0.14–0.90) (Table 2). Nevertheless, the Bland and Altman comparison showed large limits of agreement for all foods, e.g., ± 173 g/day for fruits and vegetables (Additional file, Supplementary Table 2). Moderate to high correlations ranging from 0.751 to 0.931 (all *P* < 0.001) were found for all food groups (Table 2).

**Table 2:** Mean intakes and correlations for energy intake, macronutrients and selected key healthy and unhealthy foods and beverages estimated by 24 h telephone dietary recalls and RiksmatenFlex (*n* = 52)

		Mean (SD)		Difference between means^a^	Correlation coefficient^b^			
		24 h	Riksmaten Flex	*p* value		*r*	*p* value	
Energy intake (kJ)		9427 (1817)	9432 (1936)	0.978		0.727	< 0.001	
Protein (E%)		15.0 (2.6)	14.7 (2.3)	0.322		0.707	< 0.001	
Fat (E%)		41.5 (5.3)	40.5 (5.4)	0.151		0.612	< 0.001	
Carbohydrates (E%)		41.3 (5.5)	42.5 (5.5)	0.053		0.708	< 0.001	
***Healthy foods***								
Fruit and vegetables (g/day)		426 (149)	436 (161)	0.382		0.840	< 0.001	
Fish and shellfish (g/day)		33.8 (47.2)	32.0 (40.2)	0.900^c^		0.931^d^	< 0.001	
Nuts and seeds (g/day)		9 (11)	7 (8)	0.230^c^		0.827^d^	< 0.001	
***Unhealthy foods***								
Red meat (g/day)		39 (44)	36 (34)	0.717^c^		0.912^d^	< 0.001	
Processed meat (g/day)		28 (33)	26 (32)	0.223^c^		0.852^d^	< 0.001	
Sweet and savory treats (g/day)		70 (46)	82 (60)	0.170^c^		0.751^d^	< 0.001	
Sugar sweetened beverages (g/day)		107 (177)	114 (182)	0.440^c^		0.843^d^	< 0.001	
***Other beverages***								
Artificially sweetened beverages (g/day)		78 (159)	79 (176)	0.831^c^		0.913^d^	< 0.001	
Fruit juice (g/day)		95 (108)	97 (101)	0.136^c^		0.924^d^	< 0.001	

E%, percentage of total daily energy intake; SD, standard deviation

^a^ Analyzed with parametric test (paired samples *t* test) or non-parametric test (Wilcoxon Signed Ranks Test)

^b^ Analyzed with parametric test (Pearson Correlation) or non-parametric test (Spearman correlation)

^c^ Wilcoxon Signed Ranks Test

^d^ Spearman correlation coefficient

Average intake for the SHEI score and its components from 24 h telephone dietary recalls and RiksmatenFlex are shown in Table 3. The average SHEI score assessed with 24 h telephone dietary recalls (6.44 [SD 1.01]) was not significantly different from that of RiksmatenFlex (6.60 [SD 0.99]) (*P* = 0.066). Although, a small mean difference in average SHEI score (+ 0.16 units for RiksmatenFlex versus 24 h telephone dietary recalls), the Bland and Altman plot revealed relatively wide limits of agreement of ± 1.2 units (Additional file, Supplementary Figure IV). Finally, a high correlation (*r* = 0.815, *P* < 0.001) between SHEI score assessed with the two methods was observed (Table 3). Sucrose (E%) was the only component of the SHEI score that differed between the methods (average: -1.3 E%; *P* < 0.001).

**Table 3:** Mean intakes and correlations for the Swedish Healthy Eating Index (SHEI) score and its components estimated by 24 h telephone dietary recalls and RiksmatenFlex (*n* = 52)

	Mean (SD)		Difference between means^a^	Correlation coefficient^b^	
	24 h	RiksmatenFlex	*p* value	*r*	*p* value
SHEI score	6.44 (1.01)	6.60 (0.99)	0.066	0.815	< 0.001
***Individual components of the SHEI score***					
Fruit and vegetables (g/day)	426 (149)	436 (161)	0.382	0.840	< 0.001
Wholegrain (g/10 MJ)	42.6 (29.9)	44.6 (21.5)	0.560	0.549	< 0.001
Fiber (g/MJ)	2.54 (0.72)	2.59 (0.65)	0.181^c^	0.794^d^	< 0.001
Fish and shellfish (g/day)	33.8 (47.2)	32.0 (40.2)	0.900^c^	0.931^d^	< 0.001
PUFA (E%)	5.9 (1.8)	5.8 (1.9)	0.455^c^	0.609^d^	< 0.001
MUFA (E%)	15.3 (2.2)	15.4 (2.9)	0.729	0.533	< 0.001
SFA (E%)	16.7 (3.5)	16.1 (3.0)	0.062	0.732	< 0.001
Red and processed meat (g/week)	473 (357)	438 (291)	0.275	0.771	< 0.001
Sucrose (E%)	7.6 (3.3)	8.9 (3.8)	< 0.001^c^	0.859^d^	< 0.001

E%, percentage of total energy intake; MJ, mega joule; MUFA, monounsaturated fat; PUFA, polyunsaturated fat; SD, standard deviation; SFA, saturated fat; SHEI, Swedish Healthy Eating Index

^a^ Analyzed with parametric test (paired samples *t* test) or non-parametric test (Wilcoxon Signed Ranks Test)

^b^ Analyzed with parametric test (Pearson Correlation) or non-parametric test (Spearman correlation)

^c^ Wilcoxon Signed Ranks Test

^d^ Spearman correlation coefficient

## Discussion

This is the first study to investigate validity of dietary intakes obtained with RiksmatenFlex in pregnant women. We found that the mean difference between energy intake from RiksmatenFlex and corresponding TEE obtained from the reference standard, the DLW method, was only − 237 kJ/24 h (-2.3%). Even though there are, to the best of our knowledge, no sharp cut-offs when applying the Bland and Altman method, such a small mean difference in energy intake should be considered an acceptable agreement and it is also in accordance with Lombard et al. (mean difference: 0–10%, acceptable agreement) [28]. For comparison, a review of 59 studies in adults by Burrows et al. reported that average energy intake was underestimated by 1–47% for traditional dietary methods [9]. Validation studies with DLW are scarce in pregnant women, however, Svensson et al. previously reported that a short dietary questionnaire underestimated energy intake by 21% on average in pregnant women [29]. Also, another study found that an image-based method underestimated energy intake compared to DLW by 37% in a pregnant population with obesity [30]. Thus, even though we found a tendency that lower energy intake levels were underestimated, and higher levels overestimated, RiksmatenFlex performed relatively well considering the evidence base for traditional methods as well as newer online dietary methods regarding its ability to capture average energy intakes.

Our results also indicated that RiksmatenFlex captured average intake of macronutrients as well as unhealthy and healthy foods similar to 24 h telephone dietary recalls. Moreover, the average SHEI score did not differ between RiksmatenFlex and 24 h telephone dietary recalls, indicating that RiksmatenFlex accurately can capture adherence to Swedish food-based dietary guidelines. Only one component of the SHEI score, sucrose intake (E %), was overestimated by RiksmatenFlex versus 24 h telephone dietary recalls, however, the difference was relatively small (1.3 E %). We can only speculate about the reason for this difference, but possible explanations include that women reported portion sizes for sweets and similar foods differently for the two methods. Validation studies of dietary intake through digital assessment methods in pregnancy are limited. However, a previous validation study of online 24 h dietary recalls found that the method was valid at a group level assessing energy and most nutrients when compared against a 3 day food record [31]. Further, image-based assessment methods have shown agreement when validated against 24 h dietary recalls in terms of energy as well as macro and micro nutrients [32].

The method RiksmatenFlex has previously been validated in adolescents against estimated energy expenditure from accelerometer data, biomarkers and comparison with 24 h dietary recalls [14]. In contrast to this study, RiksmatenFlex tended to overestimate average energy intake compared to estimated energy expenditure in this population [14]. This might be explained by that estimated energy expenditure was derived from accelerometer data. Accelerometry is not considered a reference standard to validate energy intake and it is well-known that accelerometers cannot capture all types of physical activity, which might be an explanation for lower estimated energy expenditure compared to energy values obtained with RiksmatenFlex. Similar to us, Lindroos et al. found that energy intakes tended to be over-reported at higher intake levels [14]. Further, RiksmatenFlex did in comparison to 24 h dietary recalls and biomarkers, capture intake of energy, fruit, vegetables and wholegrain in a comparable way in adolescents [14]. These findings are in line with our results, showing no difference in the intake of unhealthy or healthy foods and we also found that average energy intake was similar to average TEE.

We found that the limits of agreement were wide for all investigated dietary variables (energy, macronutrients, key foods and SHEI scores). To illustrate, the limits of agreement corresponded to as much as 40% of energy intake and intakes of fruits and vegetables, which limits use at an individual level. However, it is also important to note that this is commonly seen in validation studies of dietary methods in various populations including children, adults and pregnant women [14, 29, 33–35]. It is also expected as the day-to-day variation in energy intake is larger than the corresponding variation in TEE [36]. Still, further research is required in order to improve the accuracy of RiksmatenFlex and other dietary assessment methods at an individual level.

This study has several strengths. We used the DLW method to validate energy intake, which is considered the reference method [37]. We assessed dietary intake from RiksmatenFlex, 24 h telephone dietary recalls and TEE from the DLW method during the same time period, ensuring comparability between estimates. Further, the 24 h telephone dietary recalls were performed to match the days for when participants recorded their diet in RiksmatenFlex, making sure that we validated intakes from the same days and one weekend day was also included. Given that the days were matched, we cannot exclude the possibility of increased reporting accuracy if participants were affected by what earlier had been reported for that day with the other assessment method, thus there may be a risk that our observed correlation coefficients for the two dietary assessment methods are overestimated. However, in this context it is relevant to note that we also observed a strong correlation between energy intake using RiksmatenFlex and TEE using the DLW method where the methods do not share common errors [38]. Also, the order of completion of the two assessment methods was random and given that we compared three days of data and that day-to-day variation can be large, we believe this was a suitable way to validate intakes from RiksmatenFlex. Finally, we validated the SHEI score obtained from RiksmatenFlex as a measure of participants’ adherence to national food-based dietary guidelines. The SHEI score was used as a measure of participants’ diets in the HealthyMoms trial, and it was also one of the outcomes we observed an intervention effect on. There are also limitations to this study. We used 24 h telephone dietary recalls for validation of intakes of macronutrients, key unhealthy and healthy foods and the SHEI score. The 24 h recall method is not a reference method for these variables and it should be noted that both the validation method, i.e., 24 h telephone dietary recalls and RiksmatenFlex are based on self-report and share common errors, which could result in an overestimated correlation coefficient [38]. However, the 24 h recall method is a well-accepted method to assess current dietary intake and has, compared to other dietary assessment methods, been associated with less misreporting of dietary intake [9]. Moreover, we had a relatively small sample size with a relatively high educational level which might limit generalizability of our result. Furthermore, our results need to be confirmed in pregnant populations also including later stages of pregnancy and more women with obesity. Nevertheless, in this context it is important to note that the women covered a wide range in baseline characteristics including BMI as well as TEE and dietary variables. Finally, data collection was performed prior to any randomization in the HealthyMoms trial and hence participants had not taken part of any intervention content (e.g., recommendations for physical activity and diet) that might have affected their diet or physical activity levels.

Through this study we have extended the validity of RiksmatenFlex to also include pregnant women. The validation of RiksmatenFlex further strengthens the result obtained in the HealthyMoms trial where we observed a beneficial effect on diet, more specifically on the SHEI score [15], which we found to accurately be captured through RiksmatenFlex compared to 24 h telephone dietary recalls. Our findings suggest that RiksmatenFlex can be used as a dietary assessment tool in other interventions targeting pregnant women as well as in dietary surveys to capture dietary intake on a national level.

## Conclusion

We found that RiksmatenFlex accurately captured average intakes of energy, key foods (e.g., fruits and vegetables, red meat and processed meat, sweetened beverages) and adherence to food based dietary guidelines (SHEI score) in pregnant women. This validation study extends previous validation results in adolescents and supports that RiksmatenFlex may be a useful tool for national surveys and intervention studies targeting pregnant women.

### Electronic supplementary material

Below is the link to the electronic supplementary material.

**Additional file.** Compilation of supplementary tables and figures. **Supplementary Table I:** Description of the Swedish Healthy Eating Index (SHEI) score. **Supplementary Table II:** Comparison of intake of macronutrients and key healthy and unhealthy foods between RiksmatenFlex and 24 h telephone dietary recalls in accordance with the Bland and Altman procedure. **Supplementary Figures I-IX:** Bland and Altman plots and scatterplots for the comparison of macronutrients, energy and Swedish Healthy Eating Index score for RiksmatenFlex versus the 24 h dietary recalls, *n* = 52).

## Data Availability

The data that support the findings of this study are not openly available due to reasons of sensitivity and are available from the corresponding author upon reasonable request. Data are located in controlled access data storage at Karolinska Institutet.

## Abbreviations

DLW: Doubly labelled water
E%: Energy percent
GWG: Gestational weight gain
SD: Standard deviation
SHEI: Swedish Healthy Eating Index
TEE: Total energy expenditure
