# Supplementary material for: Validation of an web-based dietary assessment tool (RiksmatenFlex) against doubly labelled water and 24 h dietary recalls in pregnant women
Source: Nutr J. 2024 Jul 30;23:85. doi: 10.1186/s12937-024-00987-5 (PMC11287942; doi:10.1186/s12937-024-00987-5)
Supplement: Supplementary file 1 — Additional file. Compilation of supplementary tables and figures. Supplementary Table I: Description of the Swedish Healthy Eating Index (SHEI) score. Supplementary Table II: Comparison of intake of macronutrients and key healthy and unhealthy foods between RiksmatenFlex and 24 h telephone dietary recalls in accordance with the Bland and Altman procedure. Supplementary Figures I-IX: Bland and Altman plots and scatterplots for the comparison of macronutrients, energy and Swedish Healthy Eating Index score for RiksmatenFlex versus the 24 h dietary recalls, n = 52). [file 12937_2024_987_MOESM1_ESM.pdf]

Additional files

Validation of an Web-Based Dietary Assessment Tool (RiksmatenFlex) against Doubly Labelled Water and 24 h Dietary Recalls in Pregnant Women

Söderström et al.

Supplementary Table 1. Recommended intake for the components of the Swedish Healthy Eating Index (SHEI) score

Components of SHEI score	Recommended intake
Fruit and vegetables	>500 g/day[a]
Fiber	25-35 g/day[b]
Wholegrain	>75 g/10MJ[c]
Fish and shellfish	45 g/d[a]
Polyunsaturated fatty acids	>7.5 E%[b]
Monounsaturated fatty acids	>15 E%[b]
Saturated fatty acids	<10 E%[b]
Red and processed meat	<500 g/week[a]
Added sugar	<10 E%[b]

Table is based on and adjusted from Moraeus L, Lindroos AK, Warensjö Lemming E, Mattisson I. (2020). Diet diversity score and healthy eating index in relation to diet quality and socio-demographic factors: Results from a cross-sectional national dietary survey of Swedish adolescents. Public Health Nutr. 2020;23(10):1754-65.

[a] Based on Swedish food-based dietary guidelines (2015)
[b] Based on the Nordic Nutrition Recommendations (2012)
[c] Based on nutrient density described by Becker W, Busk L, Mattison I et al. (2012). Råd om fullkorn 2009 – bakgrund och vetenskapligt underlag. NFA report no. 10 2012 (in Swedish). Uppsala

Supplementary Table 2. Comparison of intake of macronutrients and key healthy and unhealthy foods between RiksmatenFlex and 24 h telephone dietary recalls in accordance with the Bland and Altman procedure (n=52)

	**Mean difference (g/day)[a]**	**2SD[b]**	**ρ[c]**	**p**
Protein (E%)	-0.3	3.7	-.137[d]	.332
Fat (E%)	-1.0	9.2	.020[d]	.889
Carbohydrates (E%)	1.2	8.2	.007[d]	.963
Healthy foods				
Fruit and vegetables (g/day)	10.8	173.2	.144[d]	.308
Fish and shellfish (g/day)	-1.8	38.9	.008	.954
Nuts and seeds (g/day)	-1.9	13.7	-.334	.016[e]
Unhealthy foods				
Red meat (g/day)	-3.0	55.8	-.220	.117
Processed meat (g/day)	-1.9	30.6	-.232	.099
Sweet and savory treats (g/day)	11.5	88.5	.248	.076
Sugar sweetened beverages (g/day)	7.0	116.2	-.089	.530
Other beverages				
Artificially sweetened beverages (g/day)	1.2	120.5	.159	.261
Fruit juice (g/day)	1.8	121.2	.060	.672

E%, energy percent

Calculations in table according to Bland and Altman (Bland MJ, Altman DG. Statistical methods for assessing agreement between two methods of clinical measurement. Lancet. 1986;1(8476):307–10).

[a] Calculated as RiksmatenFlex – 24 h dietary recall
[b] Calculated as twice the standard deviation
[c] Spearman correlation coefficient
[d] Pearson correlation coefficient
[e] The regression equation was $y = -0.315x + 0.594$

Supplementary figures I-IX representing Bland and Altman plots and scatterplots for the comparison of macronutrients, energy and Swedish Healthy Eating Index score for RiksmatenFlex versus the 24 h dietary recalls (n=52).

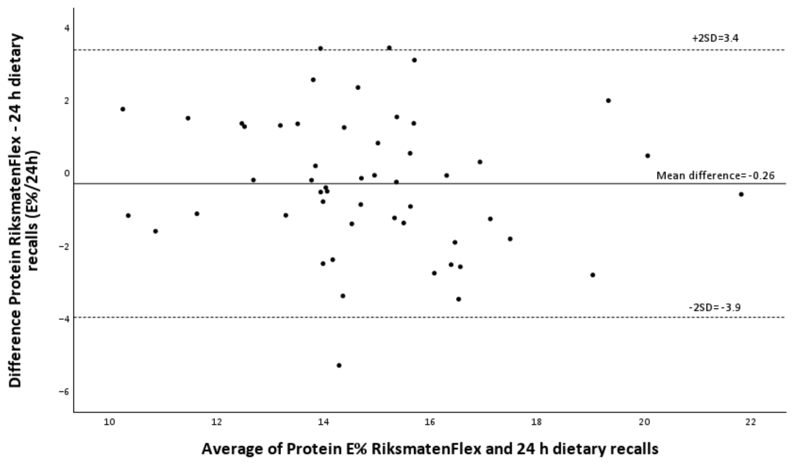

Supplementary Figure I. Bland and Altman plot for comparison of protein intake (E%) estimated using RiksmatenFlex compared to 24 h telephone dietary recalls. The mean difference between the methods was -0.26 E% per 24 h and limits of agreement (2SD) were ±3.7 E%/24 h.

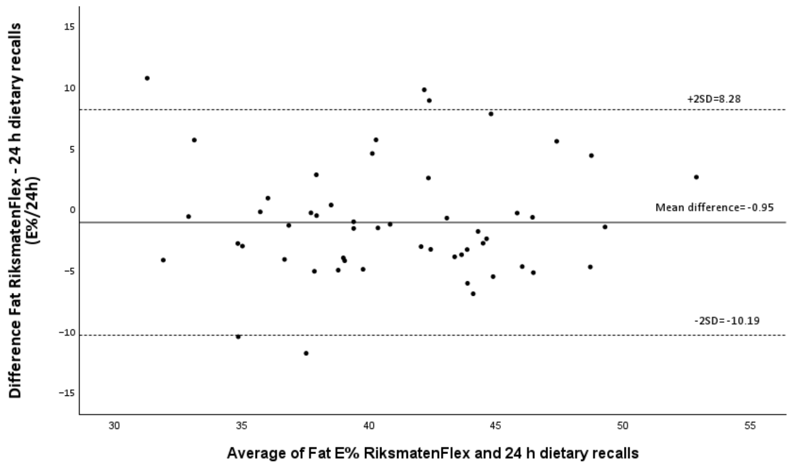

Supplementary Figure II. Bland and Altman plot for comparison of fat intake (E%) estimated using RiksmatenFlex compared to 24 h telephone dietary recalls. The mean difference between the methods was -0.95 E% per 24 h and limits of agreement (2SD) were ±9.2 E%/24 h.

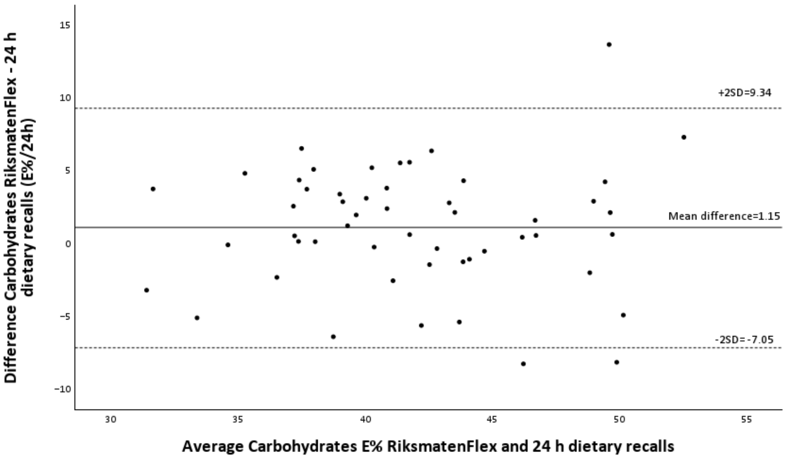

Supplementary Figure III. Bland and Altman plot for comparison of carbohydrate intake (E%) estimated using RiksmatenFlex compared to 24 h telephone dietary recalls. The mean difference between the methods was 1.15 E% per 24 h and limits of agreement (2SD) were ±8.2 E%/24 h.

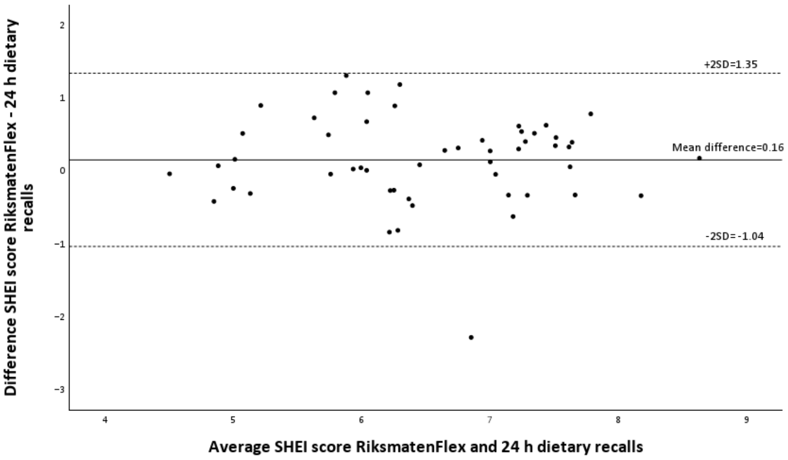

Supplementary Figure IV. Bland and Altman plot for comparison of Swedish Healthy Eating Index (SHEI) score estimated using RiksmatenFlex compared to 24 h telephone dietary recalls. The mean difference between the methods was 0.16 and limits of agreement (2SD) were ± 1.2.

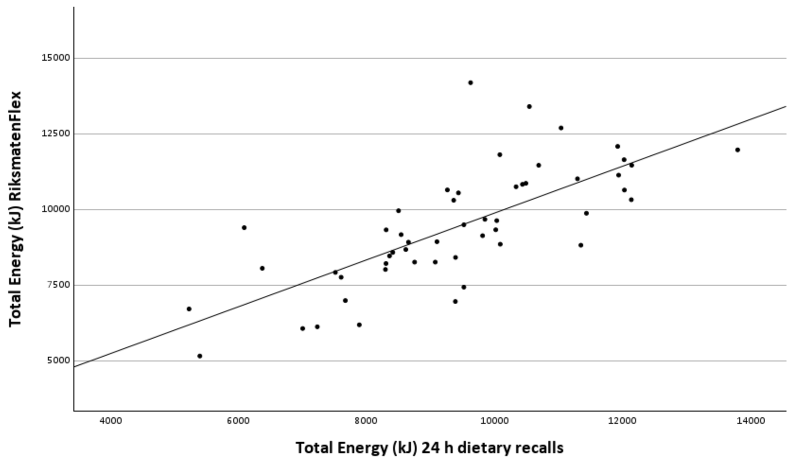

Supplementary Figure V. Scatterplot of energy intake (kJ/24 h) estimated with RiksmatenFlex against 24 h dietary recalls, r= 0.727, P<0.001.

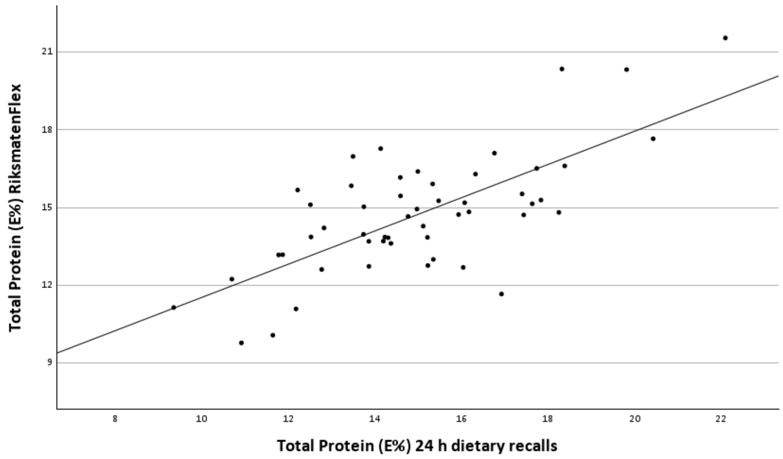

Supplementary Figure VI. Scatterplot of total protein intake (E%/24 h) estimated with RiksmatenFlex against 24 h dietary recalls, r=0.707, P<0.001.

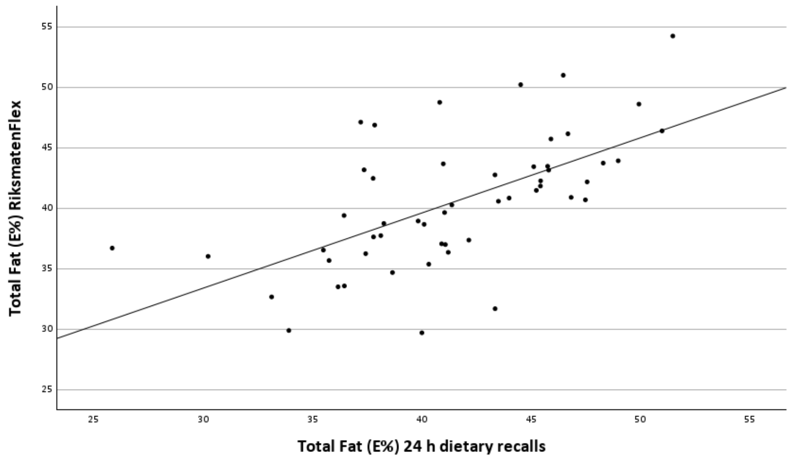

Supplementary Figure VII. Scatterplot of total fat intake (E%/24 h) estimated with RiksmatenFlex against 24 h dietary recalls, r=0.612, P<0.001.

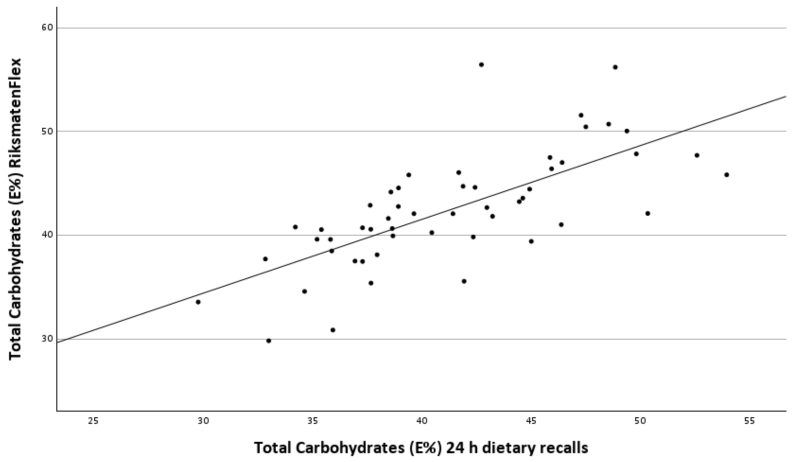

Supplementary Figure VIII. Scatterplot of total carbohydrate intake (E%/24 h) estimated with RiksmatenFlex against 24 h dietary recalls, r=0.708, P<0.001.

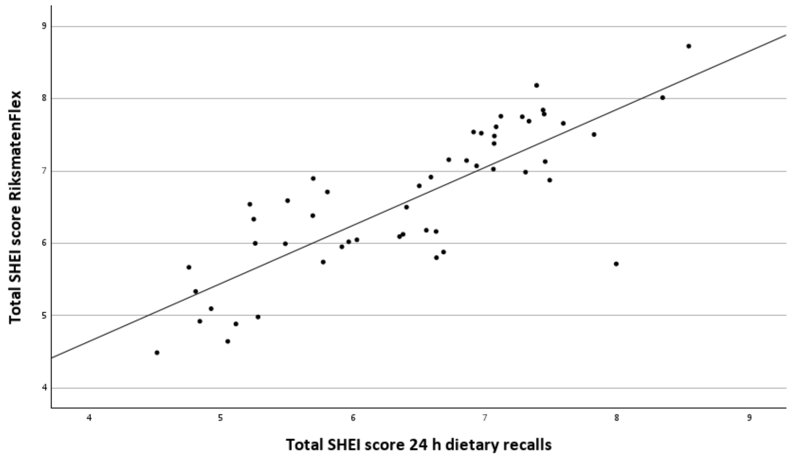

Supplementary Figure IX. Scatterplot of Swedish Healthy Eating Index (SHEI) score estimated with RiksmatenFlex against 24 h dietary recalls, r= 0.815, P<0.001.
